# Bioinformatics analysis of laryngeal squamous cell carcinoma based on the high infection rate of HPV in Northwest China

**DOI:** 10.7717/peerj.19851

**Published:** 2025-08-11

**Authors:** Fan Guo, Shurong Li, Shiqi Yan, Yuqiao Zhang, Guangke Wang, Ruixia Ma

**Affiliations:** 1 The Second Clinical Medical College, Ningxia Medical University, Yinchuan, Ningxia, China; 2 Department of Otolaryngology-Head and Neck Surgery, First Clinical College of Ningxia Medical University, Yinchuan, Ningxia, China; 3 Department of Otolaryngology, People’s Hospital of Henan Province, Zhengzhou, Henan, China; 4 Otolaryngology Head and Neck Surgery Hospital, The First People’s Hospital of Yinchuan, Yinchuan, Ningxia, China

**Keywords:** HPV, Laryngeal squamous cell carcinoma, Bioinformatics analysis, Tumor, KEGG pathway

## Abstract

**Background:**

An increasing number of studies have demonstrated that human papillomavirus (HPV) plays a crucial role in the occurrence and development of laryngeal cancer. The present study aims to identify the differentially expressed genes and pathways in HPV-positive and HPV-negative laryngeal squamous cell carcinoma (LSCC) cells for the diagnosis and treatment of HPV-related LSCC, and to determine the prevalence rate of HPV in laryngeal cancer in Northwest China.

**Methods:**

PCR-reverse dot blot was used to detect HPV genotypes in 115 LSCC patients’ paraffin sections from Jan 2022 to Jun 2024.HPV-positive TU212 cells (TU212HPV) were constructed *via* lentiviral transfection. RT-qPCR and Western blot detected mRNA and protein levels. RNA-seq and TMT sequenced gene and protein differences. DAVID database was used for Gene Ontology and pathway enrichment analyses. STRING and Cytoscape screened key genes and further analyzed pathways.

**Results:**

Among 115 patients, 64 were HPV-positive (HPV16 being the most common, 57 cases). The TU212HPV cell line was successfully constructed. RNA-seq identified 1,336 differentially expressed genes (797 upregulated, 539 downregulated). TMT found 236 differentially expressed proteins (124 upregulated, 112 downregulated). The key genes were discovered to be EGFR, CDC42, PXN, SLC2A1, GAPDH, FGF2, ICAM1, ITGB1, SFN, PGK1, and ISG15. Pathway enrichment showed involvement in neuroactive ligand-receptor interaction, Cytoskeleton in muscle cell, transcriptional misregulation in cancer, *etc*. (*P* < 0.05).

**Conclusion:**

HPV infection rate is 55.65% among laryngeal cancer patients in Northwest China. Ten key genes, namely EGFR, CDC42, PXN, SLC2A1, glyceraldehyde 3-phosphate dehydrogenase (GAPDH), FGF2, ICAM1, ITGB1, PGK1 and ISG15, as well as pathways like proteoglycans in cancer, regulation of actin cytoskeleton and HIF-1 signaling pathway are demonstrated to be of significance in the occurrence and development of laryngeal squamous cell carcinoma. PXN, ITGB1, ISG15, SLC2A1 and ICAM1 are regarded as potential therapeutic targets for HPV-positive laryngeal cancer. PXN and PGK1 are considered as potential prognostic markers for HPV-positive laryngeal cancer.

## Introduction

Head and neck squamous cell carcinoma (HNSCC) is a malignant tumor that originates from the mucosal epithelial cells of the oral cavity, pharynx, and larynx, and it occupies a significant proportion among head and neck malignancies (Johnson et al., 2020). Laryngeal squamous cell carcinoma, being the second largest subtype of HNSCC, has witnessed an upward trend in its incidence in recent years (Auperin, 2020; Jemal et al., 2007). Human papillomavirus (HPV), especially the HPV-16 and HPV-18 genotypes, plays a crucial role in the occurrence of laryngeal cancer (Liberale et al., 2023; Panuganti et al., 2021). The association is particularly evident in women, non-smokers, and younger patients (under the age of 50) (Ghosh et al., 2023; Liberale et al., 2023; Panuganti et al., 2021). This phenomenon indicates that the evaluation of HPV infection status and its typing should be given due attention in the early diagnosis and personalized treatment design of laryngeal cancer. Current research has shown that HPV genotyping is of vital importance for the early identification of intraepithelial lesions (Gluvajic et al., 2020).

The E6 and E7 genes of HPV are considered as the key driving factors in the carcinogenic process. They promote the cell cycle process and inhibit apoptosis by degrading p53 and retinoblastoma protein (Rb), thus leading to cell transformation and tumor formation (Pinkiewicz, Dorobisz & Zatonski, 2022). Although the current treatment methods for HPV-positive laryngeal cancer cases include surgical resection, chemotherapy, and radiotherapy (Pinkiewicz, Dorobisz & Zatonski, 2022), the therapeutic strategies that specifically target these core oncogenes are still in the exploratory stage.

Furthermore, with the deepening of the concept of personalized medicine for HPV-positive laryngeal cancer patients, research in related fields is gradually centering on the application of precision medicine. There is already substantial evidence suggesting that the HPV-positive status, especially the infection of the HPV-16 subtype, is associated with the improvement of the overall survival rate of patients with laryngeal squamous cell carcinoma (Panuganti et al., 2021). However, the underlying biological mechanisms still require further clarification. With the assistance of genomics and proteomics technologies, we can analyze the characteristics of laryngeal cancer tumors more meticulously, which is beneficial for a more in-depth understanding of the pathogenesis of HPV-positive laryngeal cancer and provides a theoretical foundation for devising more precise and effective treatment regimens.

## Materials and Methods

### Tumor tissue specimen

Paraffin specimens of patients with laryngeal squamous cell carcinoma with complete clinical data and pathological specimens admitted to our hospital from January 2022 to June 2024. The criteria for case inclusion and exclusion were as follows: (1) The postoperative pathological assessment confirmed the diagnosis of primary laryngeal squamous cell carcinoma, with the tumor localized to a single area; (2) complete clinical data for the patient was available; (3) the RNA content of the tissue sample met the required standards upon analysis. The collection procedures were carried out with the informed consent of all patients under a protocol approved by The First People’s Hospital of Yinchuan. Each tissue sample acquired from patients was accompanied by a definitive pathological diagnosis. The study was approved by the Ethics Committee of Yinchuan First People’s Hospital (KY-2024-017) and written informed consent was obtained from all subjects.

### PCR-reverse dot blot hybridization

HPV genotyping was detected by PCR *in vitro* amplification and DNA reverse dot hybridization. By using the specific primers designed with the genetic characteristics of HPV, the target fragments of 23 HPV genotypes can be amplified, and the 23 HPV genotypes can be detected, including 17 high-risk ones: HPV16, 18, 31, 33, 35, 39, 45, 51, 53, 56, 58, 59, 66, 68, 73, 82; six low-risk types: HPV6, 11, 42, 43, 81, 83. Then, the amplified products were hybridized with the typing probes fixed on the membrane strip, including 17 high-risk types and six low-risk types, and the infection of these HPV genotypes was determined according to the presence or non-presence of hybridization signals. Results: According to the location of blue spots on the membrane strip, the genotype information marked at the corresponding location was read: If blue spots appeared at only one genotype, it was a single infection of the corresponding genotype; If blue spots appear at multiple genotypes, it is a mixed infection of the corresponding genotypes. Positive judgment value: 120 clinical HPV-negative specimens were detected, and the gray value (int) of the detection results was calculated, and the positive judgment value was 8.8 int.

### Cell culture and treatment

The LC cells TU212 (iCell-h220) was cultivated in IMDM (iCell-0008) appended with 10% fetal bovine serum (FBS, Gibco, Waltham, MA, USA), penicillin/streptomycin (Gibco, Waltham, MA, USA), and humidity of 95% air and 5% CO_2_ at 37 °C.

### Generation of cells stably expressing HPV16E7

To gain a more targeted and in-depth understanding of the functions of E7, we decided to transfect only E7 in this experiment. TU212 cells were seeded in 6 cm culture dishes at a density of 1.5 × 10^6^ cells per dish and grown until they reached approximately 90% confluence. The recombinant plasmid pCDH-CMV-T2A-EGFP-EF1A-Puro carrying the HPV16E7 gene was then stably transfected into the cells using Lipofectamine 3000 (Invitrogen, Waltham, MA, USA). Twenty-four hours post-transfection, puromycin (2 μg/mL; Invitrogen, Waltham, MA, USA) was added to the culture medium to select for stable HPV16E7-expressing cells (TU212HPV) over a period of 2 weeks. The successful establishment of stable expression was confirmed by RT-qPCR and Western blot analysis.

### Reverse transcription‑quantitative polymerase chain reaction (RT‑qPCR)

Total RNA was isolated from TU212HPV cells and TU212 cells containing the empty plasmid (TU212CON) using Trizol Reagent (Thermo Fisher Scientific, Waltham, MA, USA). The RNA sample is mixed with the DNase I buffer and incubated at 37 °C for 15 min. After that, the DNase reaction is terminated by using EDTA. The SuperScript™ VILO™ cDNA Synthesis Kit (Thermo Fisher Scientific, Waltham, MA, USA) was applied to reverse the overall RNA into cDNA according to the instructions. Following that, the mRNA level of HPV16E7 was detected using RT-qPCR. The experiment employed PowerTrack™ SYBR Green Master Mix for qPCR (A46109; Thermo Fisher Scientific, Waltham, MA, USA) and was conducted on the 7900HT Fast Real-Time PCR System (Applied Biosystems, Waltham, MA, USA). ACTB (β-actin) served as the reference gene for data normalization. The changes in the HPV16E7 mRNA level were calculated using the 2^−∆∆Ct^ method. The primer sequences of HPV16E7and β-actin were offered:

β-actin

Forward Primer: 5′-GGCATCGTGATGGACTCCG-3′

Reverse Primer: 5′-GCTCGTCTTCACGGTTCCAT-3′

HPV16E7

Forward Primer: 5′-ATGACAGCTCAGAGGAGGAGGATG-3′

Reverse Primer: 5′-AACCGAAGCGTAGAGTCACACTTG-3′

### Western blot (WB) analysis

The amount of 50 µg of protein was isolated by using SDS-PAGE gel electrophoresis (G2044; Servicebio, Wuhan, China) and shifted to polyvinylidene difluoride (PVDF) membranes (ISEQ00010; Millipore, Billerica, MA, USA). The protein-free rapid blocking buffer (P30500; NCM Biotech, Suzhou, China) was used for membrane sealing for 15 min. The membranes were then incubated with the antibody at 4 °C overnight: Anti-E7 (1:3000, ab20191; Abcam, Cambridge, UK) and anti-tubulin (1:2000, ab179503; Abcam, Cambridge, UK) and the secondary antibody at room temperature for 1 h. The protein band was observed under the HRP ECL system (P10100; NCM Biotech, Suzhou, China), and its grey value was calculated by ImageJ version1.4 (National Institutes of Health, Bethesda, MD, USA).

### Transcriptional-level differential gene expression analysis

A total of 20,263 genes were detected. Next is the transcriptional-level differential gene expression analysis. The gene expression values of the transcripts were calculated by StringTie (version 1.3.3b). To identify the differentially expressed genes (DEGs) between two samples, DESeq2 (Love, Huber & Anders, 2014) (version 1.12.4) was employed. A gene was considered to be significantly differentially expressed under two conditions: the q-value was ≤0.001 and the absolute value of the FoldChange was ≥2. When the normalized expression of a gene was zero in both samples, its expression value was adjusted to 0.01. This adjustment was necessary because a value of 0 cannot be properly depicted on a log-scale graph. Additionally, if the normalized expression of a specific gene in both libraries was less than 1, this gene was not included in the subsequent differential expression analysis.

### Proteomics quantitative analysis: TMT-based differential protein expression study

ProteomeDiscoverer (v.2.4) was used to search all of the raw data thoroughly against the *Homo sapiens* Uniprot database. Database search was performed with Trypsin digestion specificity. Alkylation on cysteine was considered as fixed modifications in the database searching. For protein quantification method, TMT/ITRAQ was selected. A global false discovery rate (FDR) was set to 0.01 and protein groups considered for quantification required at least 1 peptide. In this study, mass spectrometry was performed on six samples. A total of 6,971 proteins were identified, among which 6,858 proteins had quantitative information. The thresholds of fold change (>1.2 or <0.833) and *P*-value < 0.05 (derived from t tests performed in R with FDR correction *via* the Benjamini-Hochberg (BH) strategy) were used to identify differentially expressed proteins (DEPs).

### Functional analysis of differentially expressed genes

Functional enrichment analyses using Gene Ontology (GO) and KEGG were carried out to find significantly enriched GO terms or metabolic pathways in differentially expressed genes (DEGs). GO, an international gene function classification system, annotates gene functions *via* Molecular Function, Cellular Component, and Biological Process. The KEGG database uses the hypergeometric test to identify enriched metabolic or signal transduction pathways in DEGs against a reference genome. GO terms and KEGG pathways with a false discovery rate (*P*-value) < 0.05 are considered significant. The DAVID (Sherman et al., 2022) online database was used to analyze the biological, cell-related, and molecular functions of DEGs through GO and KEGG enrichment pathways.

### PPI network construction and module analysis

The list of identified proteins was evaluated using version 12.0 of the STRING database (https://cn.string-db.org) to predict the identified proteins. The interaction score was set to a minimum required interaction score: 0.400. *Homo sapiens* was selected as the organism. Cytoscape 3.10.3 is a freely available platform for network visualization and analysis (Cytoscape_v3.10.3). PPI network construction in Cytoscape requires each protein in the input file to have the same identifiers. Therefore, UniProt ID mapping with Cytoscape was applied to standardize the identifiers. The interactors in the network were carefully evaluated to identify potential interactions between the nodes.

### The Cancer Genome Atlas (TCGA) database expression trend verification

To further validate the expression differences of key genes in human papillomavirus (HPV)-positive head and neck squamous cell carcinoma (HNSCC) tissues, HPV-negative HNSCC tissues, and normal tissues, and to conduct survival analysis, we utilized the UALCAN data analysis tool (http://ualcan.path.uab.edu/) to query The Cancer Genome Atlas (TCGA) database. Specifically, we obtained data from 80 HPV-positive HNSCC tissue samples, 434 HPV-negative HNSCC tissue samples, and 44 normal tissue samples. Through these data, we were able to systematically evaluate the expression patterns of key genes across different sample types and their impact on patient survival. Additionally, to more broadly validate the expression differences of these key genes between tumor tissues and normal tissues, we also employed the GEPIA2 online tool (http://gepia2.cancer-pku.cn/#index). GEPIA2 provides a convenient platform that enables researchers to directly investigate publicly available cancer transcriptome data, thereby offering additional support and validation for our findings. This approach not only helps confirm the expression characteristics of candidate genes under different pathological conditions but also provides valuable insights into how HPV status influences the biological behavior of HNSCC. By integrating resources from multiple public databases, our study aims to offer a more comprehensive understanding of the molecular mechanisms and potential therapeutic targets in HNSCC.

### Statistical analysis

Statistical analyses were performed using GraphPad Prism 10.3.0 software. The groups’ comparison and multiple groups’ comparison was analyzed by *t*-test and one-way ANOVA following Tukey’s test, respectively. All experiments were performed in triplicates at least. When the value of *P* < 0.05, the difference was identified as significance.

## Results

### HPV infection rate, type distribution and demographic characteristics of laryngeal cancer patients in Ningxia, China

**Figure 1:**
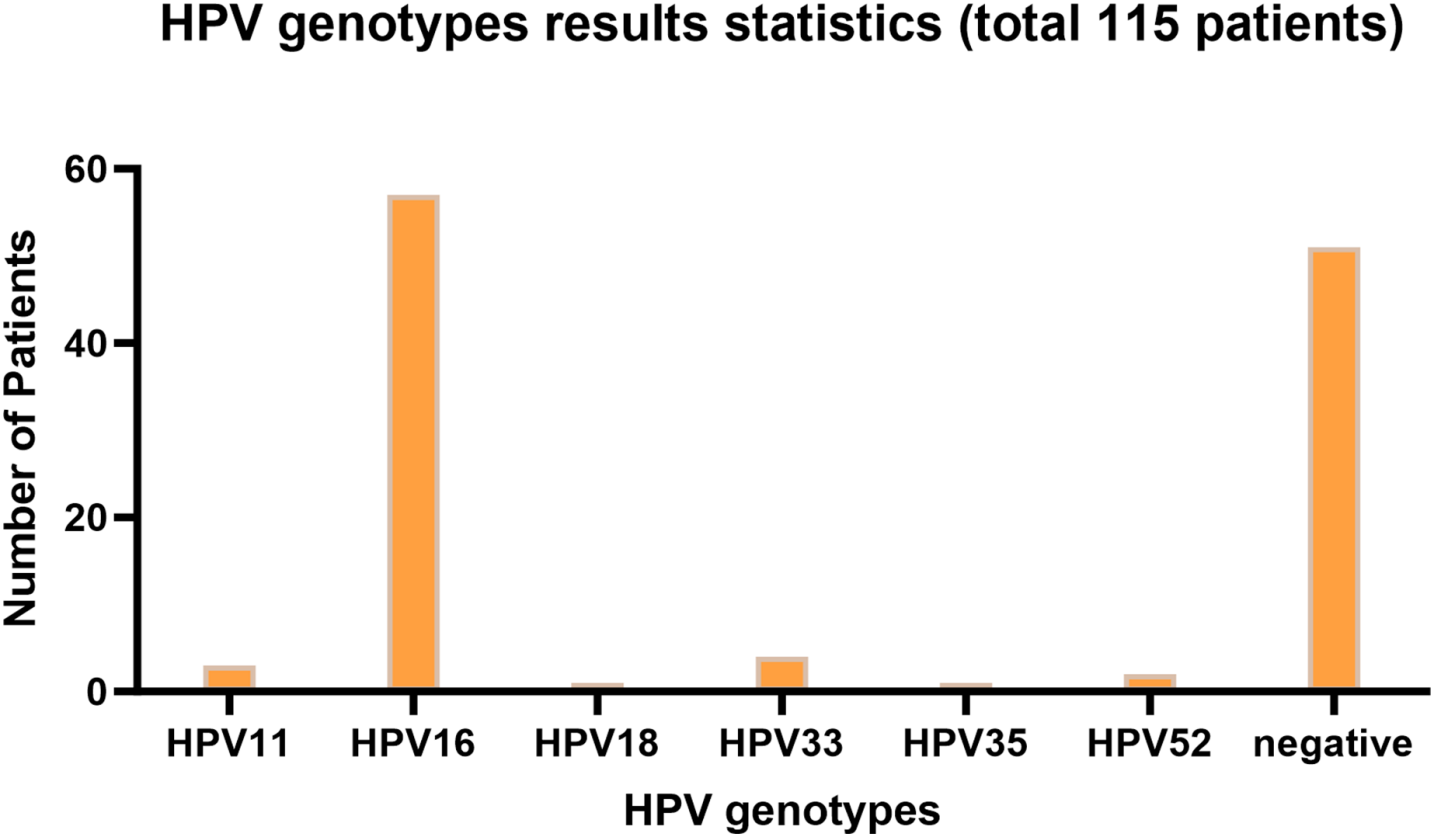
HPV genotyping statistical bar chart. The horizontal axis represents HPV genotyping, and the vertical axis represents the number of patients; The barplot was generated using Adobe Photoshop 2024.

Figure 1 presents the infection type distribution of HPV among 115 patients. The specific data indicates that the number of HPV16 infections is the highest, reaching 57 individuals, which accounts for 49.6% of the total. Subsequently, there are three patients (2.6%) with HPV11 infection, four patients (3.5%) with HPV33 infection, one patient each (0.9%) with HPV18 and HPV35 infection, and two patients (1.7%) with HPV52 infection. Among the 68 patients infected with HPV, one patient was co-infected with HPV16 and HPV18, two patients were co-infected with HPV16 and HPV33, and one patient was co-infected with HPV16 and HPV52. 51 people were not infected. It is evident from the data distribution that HPV16 is the most prevalent HPV type, while the incidences of other types of HPV are relatively low. The overall HPV infection rate of laryngeal cancer in Ningxia, China is 55.65%.

Among HPV-positive laryngeal cancer patients, ages ranged from 42 to 84 years, with median 61, first quartile 55, third quartile 67, and mean about 63.18. Females accounted for 6.25%. For age comparison between HPV-negative and -positive patients, an unpaired t test was done. Results: *P*-value 0.8232 (ns), two-tailed, t = 0.2239, df = 113, no significant difference as *P* < 0.05 not met. For gender difference analysis, a Fisher’s exact test was used. *P*-value 0.6916 (ns), two-sided, no statistical significance as *P* < 0.05 not satisfied. In conclusion, no significant differences in age or gender were detected between the two groups.

### A laryngeal carcinoma cell harboring HPV16E7 was successfully transfected

**Figure 2:**
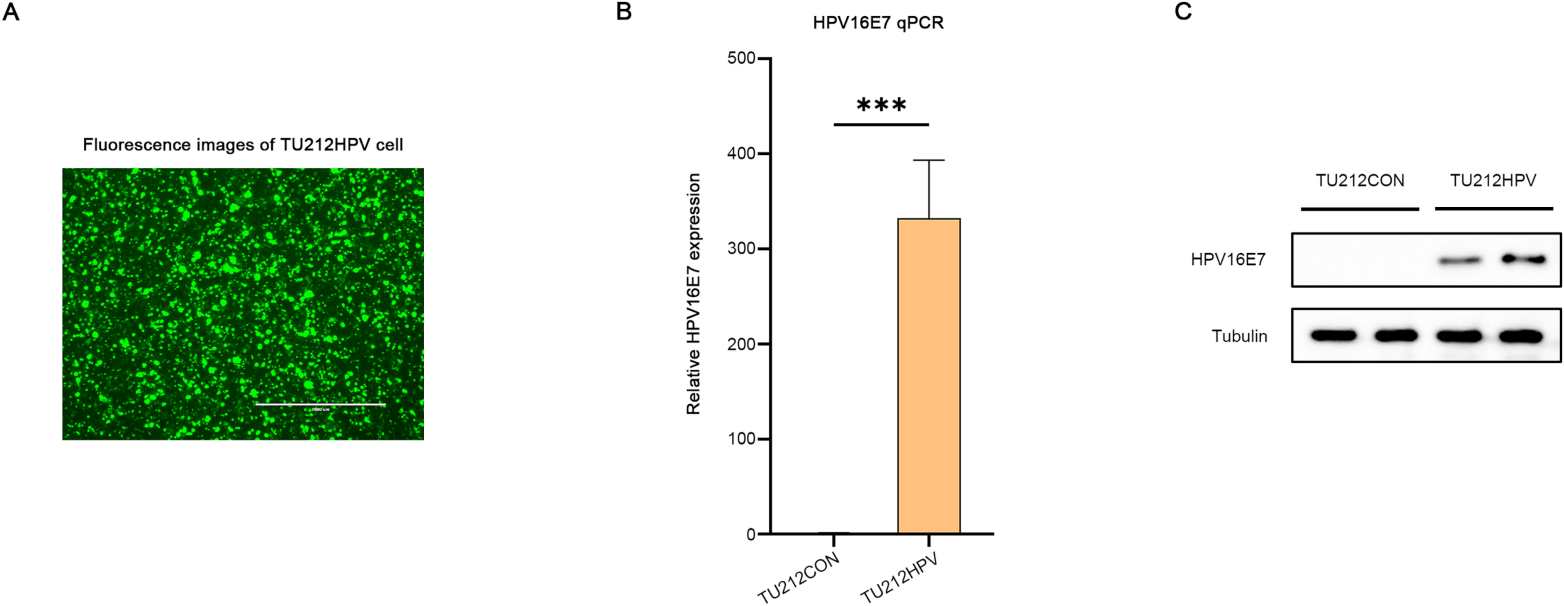
Successful transfection of HPV16E7 in TU212 cells. (A) Fluorescence image after successful transfection of TU212HPV. (B) RT-qPCR was used to detect the content of E7 mRNA in TU212HPV and TU212CON (TU212 containing an empty plasmid) (***p < 0.001). (C) WB was used to detect the content of E7 protein in TU212HPV and TU212CON. The barplot was generated using Adobe Photoshop 2024.

A laryngeal cancer cell line TU212HPV was established, which stably expresses HPV16E7. As depicted in Fig. 2, the TU212HPV cells, upon successful construction, exhibited green fluorescence when visualized under a laser scanning microscope (Fig. 2A). Moreover, RT-qPCR and Western blot assays were employed to determine the expression levels of E7 mRNA and protein (Figs. 2B, 2C). TU212CON represents the TU212 laryngeal cancer cell line carrying an empty plasmid.

## RNA sequencing data analysis

### DEGs identification

**Figure 3:**
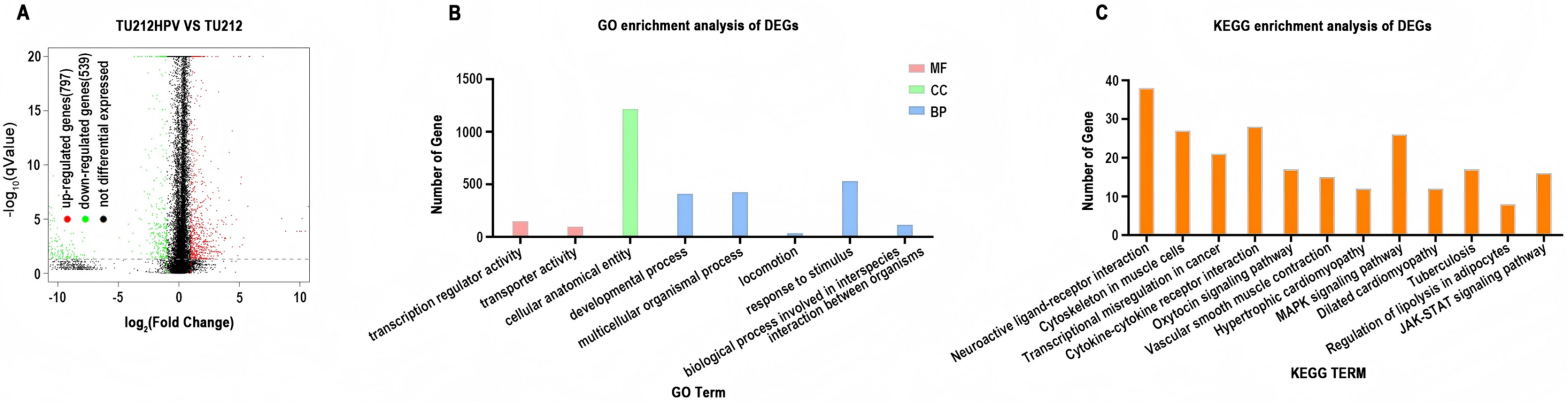
RNA sequencing data analysis. (A) Volcano plot of expression differences between the TU212HPV and TU212 groups. The horizontal axis represents the fold-change (log2 (B/A)) value of gene expression differences between different groups of samples, and the vertical axis represents the statistical significance *P*-value of gene expression changes. The smaller the *P*-value, the larger the −log10 (*P*-value), and the more significant the difference. Each point in the figure represents a gene, where red indicates up-regulated genes, green indicates down-regulated genes, and black indicates non-differential genes. (B) Bar chart of GO annotation classification for differential genes between TU212HPV and TU212; the horizontal axis represents functional classification, and the vertical axis represents the number of genes in this classification; different colors represent different classifications. Red represents molecular function, green represents cellular component, and blue represents biological process. (C) Bar chart of KEGG annotation classification for differential genes between TU212HPV and TU212; the horizontal axis represents KEGG term, and the vertical axis represents the number of genes in this term. The *P*-values of all enrichment analyses are less than 0.05.

DESeq2 (version 1.12.4) was used to determine differentially expressed genes (DEGs) between two samples. Genes were considered as significant differentially expressed if q-value ≤ 0.001 and |FoldChange| ≥ 2. Gene expression differences were visualized by volcano plot (Fig. 3A). After differential expression analysis, a total of 1,336 significantly differentially expressed genes (DEGs)were identified (including 797 up-regulated genes and 539 down-regulated genes.) in TU212HPV when compared to TU212.

### Ontology analysis of differential genes

Gene IDs of 1,336 differential genes were input into DAVID database for gene ontology analysis. It turns out that over the course of biological process (BP), differentially expressed genes are mainly involved in developmental process, multicellular organismal process, locomotion, response to stimulus and biological process involved in interspecies interaction between organisms. In terms of molecular function (MF), differentially expressed genes are mainly involved in the functions of transcription regulator activity and transporter activity (Fig. 3B).

### KEGG analysis of differential genes

Analysis using the DAVID database analysis reveals that the differentially expressed genes primarily participate in pathways such as neuroactive ligand-receptor interaction, cytoskeleton in muscle cells, MAPK signaling pathway, and cytokine-cytokine receptor interaction. Additionally, these genes are involved in pathways known to be associated with cancer development and progression, including transcriptional misregulation in cancer, cytokine-cytokine receptor interaction, MAPK signaling pathway, and JAK-STAT signaling pathway (Fig. 3C). The results of GO and KEGG analyses of the upregulated and downregulated differentially expressed genes have been placed in GO and KEGG Statistical Summary Table of the Supplemental Materials.

## Quantitative proteomic analysis

### Differential proteins identification

**Figure 4:**
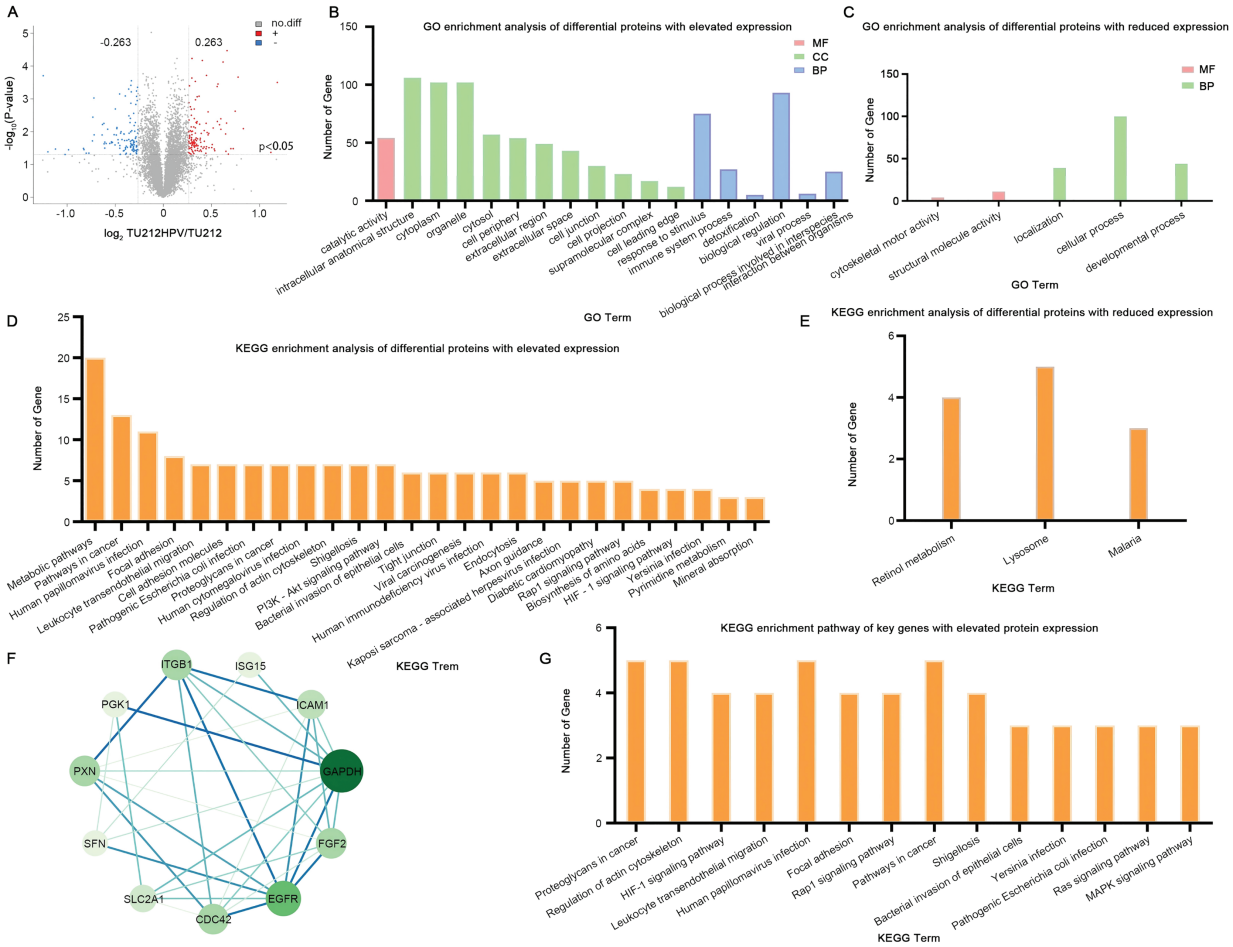
Quantitative proteomic analysis. (A) Volcano plot of TU212HPV *vs*. TU212 expression differences. Horizontal axis: fold-change (log2 (B/A)) of gene expression. Vertical axis: *p v*alue of gene expression change (−log10). Red dots: genes up-regulated in TU212HPV *vs*. TU212. Blue dots: genes down-regulated in TU212HPV *vs*. TU212. Black dots: non-differential genes. (B–C) GO annotation classification bar chart of TU212HPV *vs*. TU212 differential proteins. Horizontal axis: functional classification. Vertical axis: number of genes in each classification. Red: molecular function. Green: cellular component. Blue: biological process. (D–E) KEGG annotation classification bar chart of TU212HPV *vs*. TU212 differential proteins. The horizontal axis represents the KEGG term, and the vertical axis represents the number of genes in this term. (F) The PPI (Protein-Protein Interaction) graph of differentially expressed genes with increased expression. (G) KEGG enrichment pathways of Key Genes with up-regulated differential expression. The *P*-values of all enrichment analyses are less than 0.05.

In the quantitative proteomic analysis, the differential proteins were screened by specific criteria. Specifically, the screening was based on the quantitative protein ratios and the *P*-values of statistical tests. I chose the TMT method for protein quantification. The thresholds of fold change (>1.2 or <0.833) and *P*-value <0.05 were set to identify the differentially expressed proteins (DEPs). Eventually, a total of 236 differential proteins were detected, among which 124 were highly expressed and 112 were lowly expressed (Fig. 4A). The complete raw results of high-throughput sequencing and protein sequencing have been uploaded to the public repository Zenodo (DOI 10.5281/zenodo.15389257).

### Ontology analysis of differential proteins

A total of 124 UniProt accession numbers of highly expressed proteins were input into the DAVID database, and 126 DAVID IDs were retrieved for further gene ontology analysis. The results indicate that in biological processes, the differentially expressed proteins are mainly involved in major functions such as the response to stimulus, the biological process involved in interspecies interaction between organisms, the immune system process, detoxification, biological regulation, and the viral process. In terms of molecular functions, the differentially expressed proteins are mainly engaged in catalytic activity functions (Fig. 4B).

By the same method, 112 differential proteins with low expression were analyzed. The results showed that in biological processes, these differentially expressed proteins were mainly involved in localization, cellular process, and developmental process. At the cell component level, no enrichment was observed when *p* < 0.05. In terms of molecular function, the differentially expressed proteins were mainly involved in cytoskeletal motor activity and structural molecule activity (Fig. 4C).

### KEGG analysis of differential proteins

Utilizing the DAVID database to conduct an analysis of the differentially expressed proteins between the two groups, the resultant KEGG enrichment pathways are presented in Figs. 4D, 4E. Upon careful examination and interpretation of these figures, it becomes evident that the proteins exhibiting increased expression levels as a direct result of HPV infection possess a more profound and significant impact on the complex processes of oncogenesis and progression within laryngeal cancer cells. Their roles are likely intertwined with various molecular mechanisms and signaling pathways that contribute to the malignant transformation and advancement of the disease. Given the notable influence of these upregulated proteins, our research focus will be strategically centered and predominantly directed towards a more in-depth exploration and understanding of their functions, interactions, and potential as therapeutic targets.

### PPI network analysis

In this study, we focused specifically on the differentially expressed genes with increased expression for further analysis. To deeply investigate the interaction relationships among these genes, 124 such genes were imported into the STRING database, and a PPI network was successfully constructed. This network consisted of 124 nodes (*i.e*., target proteins) and 244 edges (representing protein-protein interactions). Among them, the degree value was employed to quantify the number of interactions between a protein and other proteins. After calculation, the average degree value of all nodes in this network was 3.94. Subsequently, with the help of Cytoscape 3.10.3 software, nodes with a degree value less than double the average degree value (7.88) were removed. Then, the obtained PPI network was re-screened to eliminate nodes with values less than the average betweenness centrality (BC), average closeness centrality (CC), and average degree value. Eventually, a PPI network composed of 11 nodes and 32 edges was obtained. The 11 key proteins were EGFR, CDC42, paxillin (PXN), SLC2A1, glyceraldehyde 3-phosphate dehydrogenase (GAPDH), FGF2, ICAM1, ITGB1, SFN, PGK1, ISG15 (Fig. 4F). Further KEGG enrichment analysis was carried out on these 11 key proteins, and a total of 14 pathways were obtained (Fig. 4G). Table S1 (Please refer to Table S1 for details.) also presents the genes enriched in each pathway. SFN was not enriched, and among the remaining 10 key proteins, CDC42 was enriched 13 times, ITGB1 was enriched 12 times, EGFR was enriched 10 times, and PXN was enriched nine times. These pathways were mainly involved in proteoglycans in cancer, regulation of actin cytoskeleton, HIF-1 signaling pathway, leukocyte transendothelial migration, and human papillomavirus infection.

Since our research was restricted to the genes with increased expression, we did not conduct the same in-depth analysis on the genes with reduced expression as described in the original text. This was due to the research focus of this project, which aimed to understand the specific roles and interactions of the upregulated genes in the context of the biological process.

## The influence of HPV status on the expression of key genes in head and neck squamous cell carcinoma and the correlation analysis with patient survival rates

**Figure 5:**
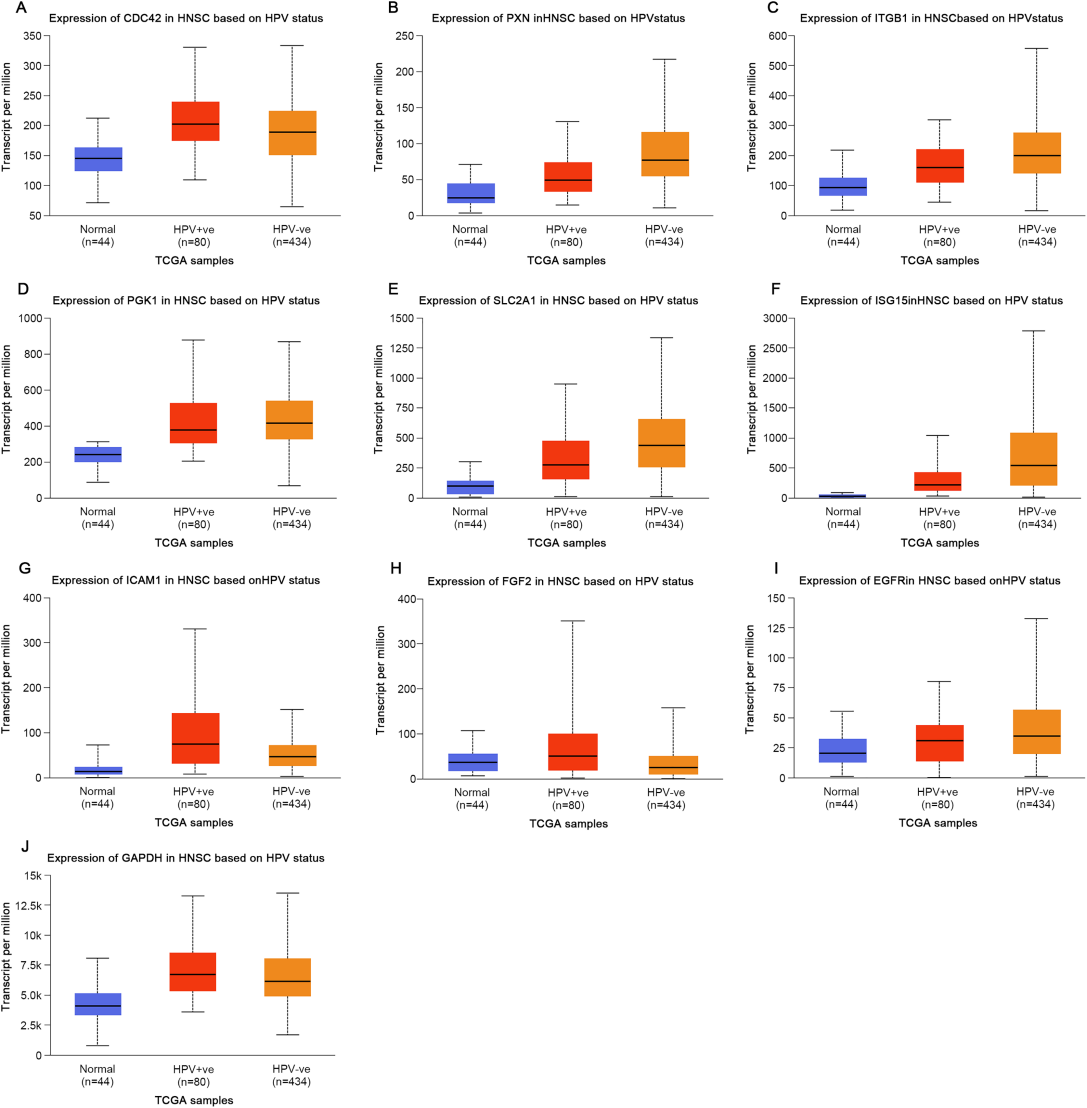
Expression of key genes in HNSC based on HPV status. (A) CDC42; (B) PXN; (C) ITGB1; (D) PGK1; (E) SLC2A1; (F) ISG15; (G) ICAM1; (H) FGF2; (I) EGFR; (J) GAPDH.

**Figure 6:**
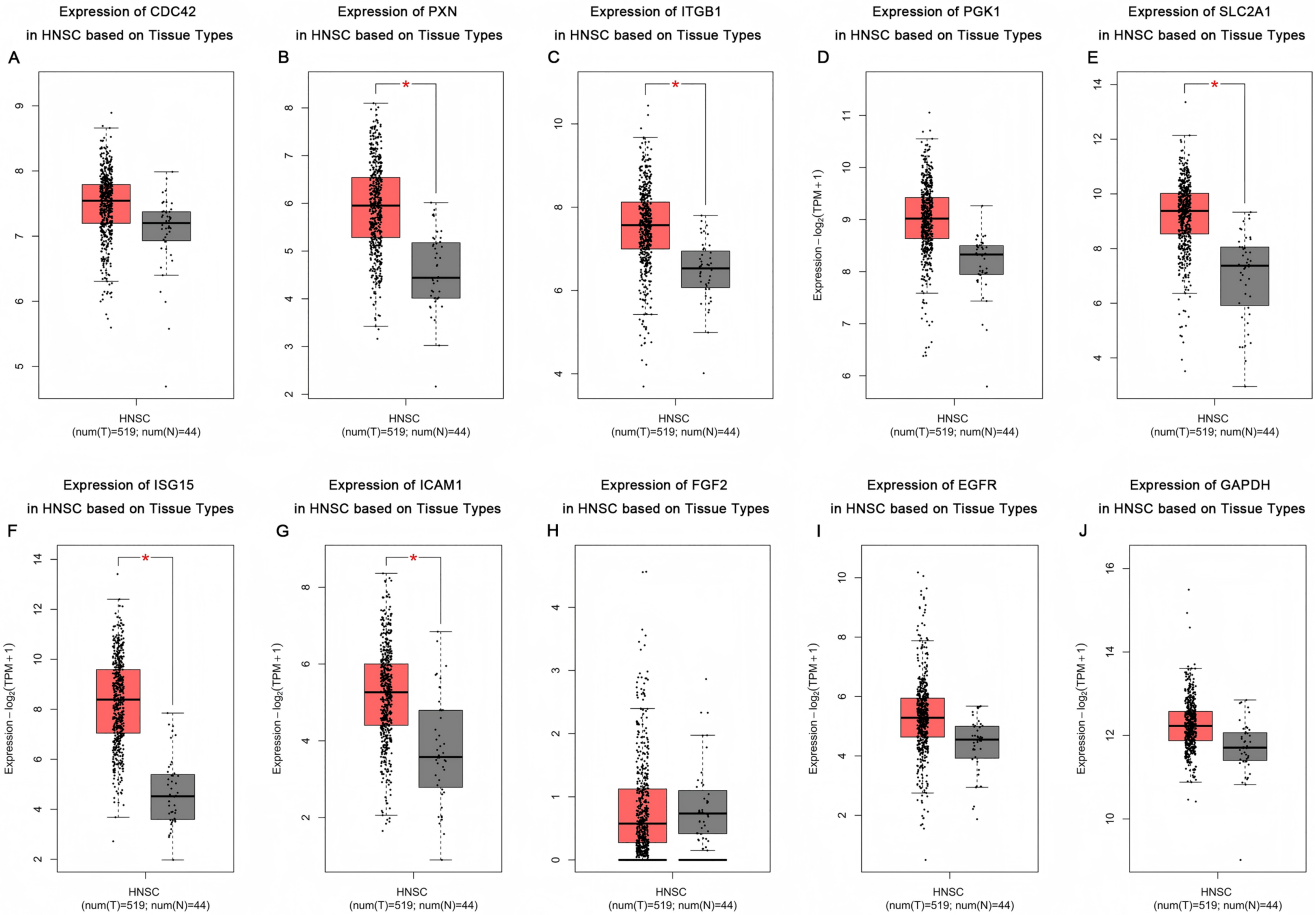
Expression of key genes in HNSC based on tissue types. Red represents tumor tissues, and gray represents normal tissues. (A) CDC42; (B) PXN; (C) ITGB1; (D) PGK1; (E) SLC2A1; (F) ISG15; (G) ICAM1; (H) FGF2; (I) EGFR; (J) GAPDH. (*p < 0.05).

**Figure 7:**
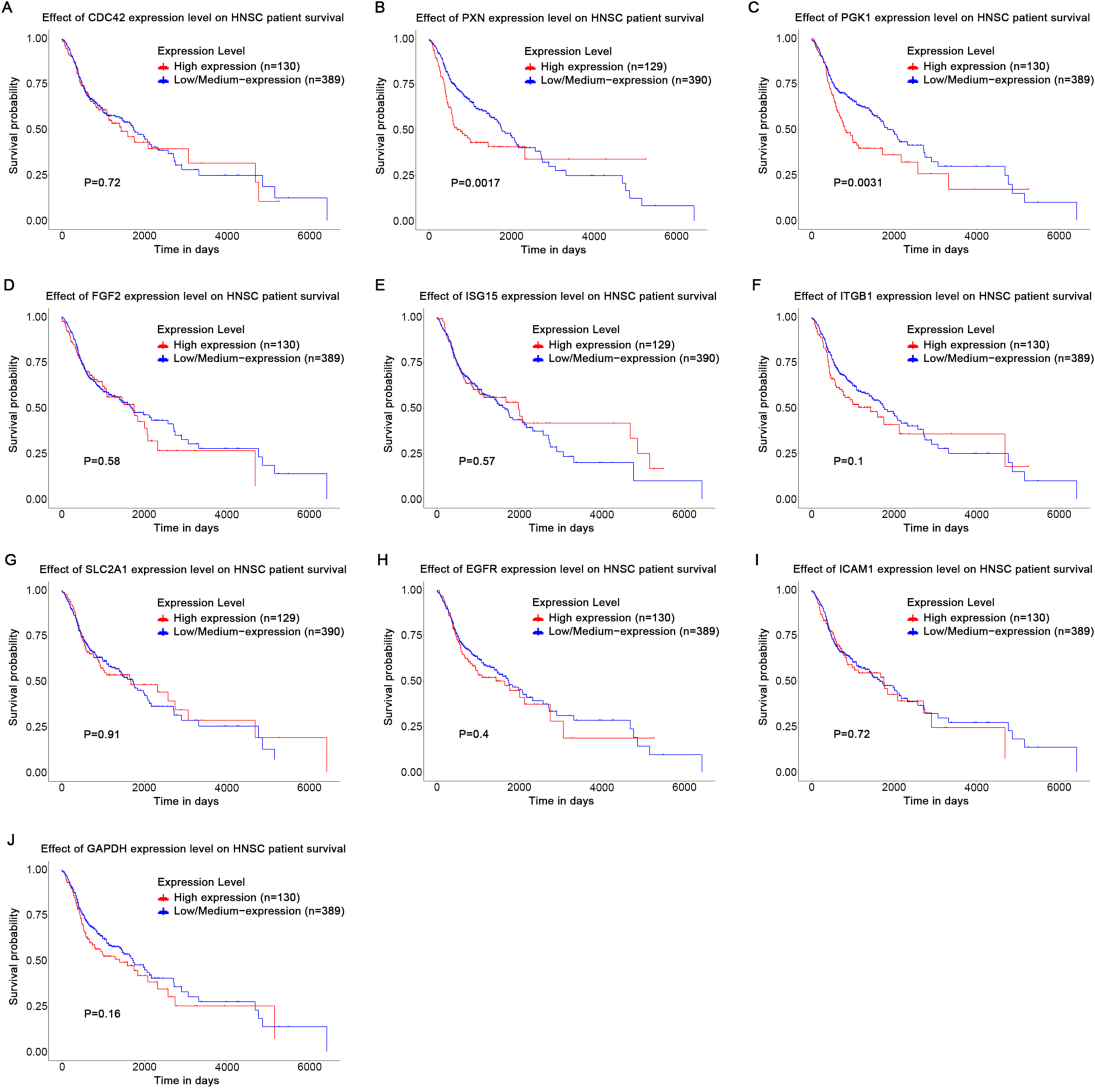
Effect of key genes expression level on HNSC patient survival. (A) CDC42; (B) PXN; (C) PGK1; (D) FGF2; (E) ISG15; (F) ITGB1; (G) SLC2A1; (H) EGFR; (I) ICAM1; (J) GAPDH.

Based on the UALCAN analyses of the TCGA databases, in HPV-positive head and neck squamous cell carcinoma (HNSCC), although the *P*-values were not statistically significant, we observed a trend toward higher expression levels of the CDC42, FGF2, ICAM1, and GAPDH genes compared to both HPV-negative and normal tissues. Although the expressions of ISG15, ITGB1, PXN, SLC2A1, PGK1 and EGFR in HPV-positive HNSCC were slightly lower than those in HPV-negative tissues, they were still higher than those in normal tissues (Fig. 5). Based on GEPIA2 analyses of the TCGA and GTEx databases, the expressions of ISG15, ITGB1, PXN, SLC2A1 and ICAM1 in HNSCC tumor tissues were considerably higher than those in normal tissues (*P* < 0.05) (Fig. 6). Additionally, further investigation using the UALCAN based on TCGA databases indicated that the expression levels of PXN and PGK1 were significantly correlated with the survival rates of HNSCC patients (*P* < 0.05) (Fig. 7).

## Discussion

In laryngeal cancer research, human papillomavirus (HPV) is of critical importance (Stumbryte-Kaminskiene et al., 2020). HPV infection is closely associated with the occurrence, development, prognosis and treatment response of laryngeal cancer, significantly modifying the tumor’s biological processes (Stumbryte-Kaminskiene et al., 2020). Key genes like CDC42, PXN, ITGB1, PGK1, SLC2A1, ISG15 and ICAM1, affected by HPV, display distinct functional alterations and complex correlations in laryngeal cancer progression. Analyzing these genes in HPV-related laryngeal cancer will provide key insights for pathogenesis, precise treatments and prognostic markers, advancing related research and clinical practice.

Paxillin is involved in cytoskeletal reorganization and the formation of focal adhesions, exerting a crucial role in the migratory and metastatic processes of cancer cells (Okada et al., 2020; Wang et al., 2020; Wu et al., 2023; Zhao et al., 2020). In the head and neck squamous cell carcinoma (HNSCC) cell models, the downregulation of paxillin contributes to the attenuation of tumor cell invasion and metastasis (Hefni et al., 2023; Wu et al., 2014). In clinical specimens of HNSCC, the overexpression of PXN mediated by HPV E6 drives tumor progression and correlates with inferior disease-free survival, recurrence-free survival, and overall survival rates (Hefni et al., 2023; Wu et al., 2014; Zhao et al., 2020). In other malignancies, elevated expression of the PXN gene is associated with poorer overall survival (Chawhan & Dsouza, 2024; Du et al., 2016; Wang et al., 2020) and also serves as an independent prognostic determinant for patient survival (Okada et al., 2020). The expression level of PXN is linked to cancer drug resistance. It is implicated in the sensitivity of colorectal cancer cell lines to cetuximab (Du et al., 2016), and in lung cancer cells, it potentially leads to cisplatin resistance *via* ERK-mediated Bcl-2 transcriptional activation (Chawhan & Dsouza, 2024). Consequently, suppression of PXN expression may represent a rational therapeutic approach to surmount tumor drug resistance. The aforementioned experimental findings furnish robust evidence suggesting that PXN not only functions as a potential therapeutic target for HNSCC but also its expression level can be exploited as a biomarker for prognostication of patient outcomes, particularly in HPV-infected cases.

The mRNA and protein levels of ITGB1 are significantly upregulated in a variety of tumor tissues (Kwon et al., 2022; Liu et al., 2020; Lv et al., 2024; Xie et al., 2021). As a part of the pathway involved in extracellular matrix-receptor interactions, ITGB1 exhibits a remarkable increase in expression in HPV-related head and neck cancer (HNC), cervical cancer (CC), and patients with OCSCC (Kwon et al., 2022; Lv et al., 2024; Park et al., 2023). Moreover, the upregulated expression of ITGB1 augments the invasiveness and migratory capacity of radioresistant OSCC cells (RROSCC) (Park et al., 2023). This indicates that ITGB1 might play a crucial role in the tumor development process. ITGB1 is also associated with a poor prognosis and lower overall survival (OS) and recurrence-free survival (RFS) (Kwon et al., 2022; Liu et al., 2020; Lv et al., 2024). In conclusion, ITGB1 plays a pivotal role in multiple tumors, and its expression level is closely related to the poor prognosis of patients. Therefore, it has the potential to serve as a therapeutic target and prognostic marker for HNSCC.

The HIF-1 signaling pathway enables cells to adapt to the hypoxic environment by regulating the expression of genes involved in multiple processes, such as PGK1 and SLC2A1. One of the hallmark features of cancer is the alteration of its metabolic pattern, which usually includes an increased rate of glycolysis, even under aerobic conditions, a phenomenon known as the Warburg effect (Mathew et al., 2024). PGK1 and SLC2A1 plays an important role in this phenomenon to promote the increased glycolytic rate of tumor cells (Han et al., 2021; Miller et al., 2024). In this study, it was found that PGK1 and SLC2A1 were highly expressed in TU212HPV cells and were enriched in the HIF-1 signaling pathway, indicating that PGK1 and SLC2A1 play important roles in maintaining the energy metabolism of laryngeal cancer cells, adapting to hypoxic conditions and promoting tumor development. Meanwhile, in head and neck squamous cell carcinoma (HNSCC), the expression levels of PGK1 and SLC2A1 were upregulated and were significantly associated with the patients’ poorer overall survival (OS), disease-free survival (DFS) and relapse-free survival (RFS) (Kunkel et al., 2003; Miller et al., 2024; Swartz et al., 2016; Wang et al., 2023). The E7 oncoprotein was also found to upregulate the expression levels of SLC2A1 protein and mRNA in lung cancer cells (Gao et al., 2021). This further emphasizes that SLC2A1 has a great potential to be a prognostic marker and a therapeutic target for HPV-positive laryngeal cancer.

ISG15 was significantly upregulated in HNSCC tissues compared with normal controls (Li et al., 2024b; Palollathil et al., 2024; Thakore et al., 2024). The tumor microenvironment was affected and the development and metastasis of tumors were promoted by ISG15 through specific signal transduction pathways (Li et al., 2024b; Qin et al., 2024). Currently, the high expression of ISG15 has been identified as an important biomarker for predicting the response to immune checkpoint inhibitors (Kunkel et al., 2003). Therefore, in HPV-positive HNSCC patients, a high level of ISG15 expression may predict a better immunotherapy effect.

In HNSCC tissues, ICAM1 expression was markedly higher than in adjacent healthy tissues and positively correlated with macrophage infiltration (Li et al., 2024a). ICAM1 is an essential mediator in macrophage-HNSCC interaction and closely related to tumor cell stemness, invasiveness and chemoresistance (Hsieh et al., 2022). The neoantigen against mICAM1 has succeeded in a mouse oral squamous cell carcinoma model (Zolkind et al., 2017). To sum up, the results strongly support ICAM1 as a potential HNSCC treatment target, given its role in enhancing immune cell recognition and clearance of cancer cells (Li et al., 2024a). Thus, ICAM1 is both a valuable biomarker and a key target for novel HNSCC treatment development.

CDC42, a small GTPase belonging to the rho subfamily, regulates signaling pathways that govern a multiplicity of cellular functions, encompassing cell morphology, migration, endocytosis, and cell cycle progression (Buckley & St Johnston, 2022; Castillo-Azofeifa et al., 2023; Chernichenko et al., 2020; Zhang et al., 2019). In the context of recurrent or metastatic oropharyngeal squamous cell carcinoma (OSCC, a form of HNSCC), CDC42 partakes in the pathologic and progressive aspects of the disease by modulating malignant tumor behavior and immune evasion (Chen, Chen & Liu, 2024). High expression levels of CDC42 independently prognosticate shorter progression-free survival (PFS) and overall survival (OS) (Chen, Chen & Liu, 2024), thereby intimating its significance as a prognostic biomarker.

My research has demonstrated that the HPV infection rate of laryngeal cancer in Northwest China is relatively higher when compared with that in European and American countries, which is in line with the conclusion drawn by Zhang et al. (2016). In laryngeal cancer, HPV type 16 is the most common type, and its infection status has no significant difference with the gender and age of laryngeal cancer patients (Brito et al., 2022).

In this experiment, only one cell line was used, and the sample size was relatively small, with only three cases in each group. For future research, we plan to include more cell lines and intend to use techniques such as qPCR to quantitatively validate the differential expression of key genes in independent patient groups (such as HPV-positive and HPV-negative laryngeal cancer tissues). In addition, functional validation experiments should be carried out. These experiments will include gene knockdown or overexpression assays, as well as assessments of cell proliferation, migration, and invasion, to further clarify the biological functions of these genes.

## Conclusions

Based on this experimental research and the above results, it can be seen that HPV is closely related to oncogenes. The HPV positive rate in Northwest China is relatively high. ISG15, ITGB1, PXN, SLC2A1 and ICAM1 are expected to become potential therapeutic targets for HPV-positive laryngeal cancer. PXN and PGK1 are expected to be potential prognostic markers for HPV-positive laryngeal cancer.

## Supplemental Information

10.7717/peerj.19851/supp-1 Supplemental Information 1 Raw images of HPV genotyping.

10.7717/peerj.19851/supp-2 Supplemental Information 2 MIQE checklist.

10.7717/peerj.19851/supp-3 Supplemental Information 3 Statistical results of HPV typing.

10.7717/peerj.19851/supp-4 Supplemental Information 4 HPV16E7 RT-qPCR raw data and calculation procedure.

10.7717/peerj.19851/supp-5 Supplemental Information 5 The genes enriched in each pathway.

10.7717/peerj.19851/supp-6 Supplemental Information 6 Nucleic acid quantification, Purity (A260A280), Yield, Amount of RNA and reaction volume.

10.7717/peerj.19851/supp-7 Supplemental Information 7 STR Genotype Detection Report of TU212 Cells.

10.7717/peerj.19851/supp-8 Supplemental Information 8 GO and KEGG Statistical Summary Table. All results of the GO and KEGG analyses performed on the differential genes and proteins.

10.7717/peerj.19851/supp-9 Supplemental Information 9 HPV16E7 WB original data. Full-length uncropped gels/blots. A photograph of the reassembled pieces to confirm that they come from the same original full-length blot. Molecular weight ladders, controls and reference samples must be visible.

## Additional Information and Declarations

### Data Availability

The following information was supplied regarding data availability:

Raw data (including HPV16E7 RT-qPCR, unprocessed images of HPV genotyping, and unprocessed Western blots) are available in the Supplemental Files.

## References

[ref-1] Auperin A (2020). Epidemiology of head and neck cancers: an update. Current Opinion in Oncology.

[ref-2] Brito C, Cossetti RD, de Souza DA, Catanha M, de Matos Monteiro P, Vidal FCB (2022). Prevalence of HPV genotypes and assessment of their clinical relevance in laryngeal squamous cell carcinoma in a northeastern state of Brazil-a retrospective study. PeerJ.

[ref-3] Buckley CE, St Johnston D (2022). Apical-basal polarity and the control of epithelial form and function. Nature Reviews Molecular Cell Biology.

[ref-4] Castillo-Azofeifa D, Wald T, Reyes EA, Gallagher A, Schanin J, Vlachos S, Lamarche-Vane N, Bomidi C, Blutt S, Estes MK, Nystul T, Klein OD (2023). A DLG1-ARHGAP31-CDC42 axis is essential for the intestinal stem cell response to fluctuating niche Wnt signaling. Cell Stem Cell.

[ref-5] Chawhan AP, Dsouza N (2024). Identifying the key hub genes linked with lung squamous cell carcinoma by examining the differentially expressed and survival genes. Molecular Genetics and Genomics.

[ref-6] Chen Y, Chen Y, Liu W (2024). Serum cell division control 42 reflects treatment response and survival profiles in recurrent or metastatic oral squamous cell carcinoma patients who receive programmed death-1 inhibitors. International Immunopharmacology.

[ref-7] Chernichenko N, Omelchenko T, Deborde S, Bakst RL, He S, Chen CH, Gusain L, Vakiani E, Katabi N, Hall A, Wong RJ (2020). Cdc42 mediates cancer cell chemotaxis in perineural invasion. Molecular Cancer Research.

[ref-8] Du C, Wang X, Zhang J, Liu X, Zhu J, Liu Y (2016). Paxillin is positively correlated with the clinicopathological factors of colorectal cancer, and knockdown of Paxillin improves sensitivity to cetuximab in colorectal cancer cells. Oncology Reports.

[ref-9] Gao ZY, Gu NJ, Wu MZ, Wang SY, Xu HT, Li QC, Wu GP (2021). Human papillomavirus16 E6 but not E7 upregulates GLUT1 expression in lung cancer cells by upregulating thioredoxin expression. Technology in Cancer Research & Treatment.

[ref-10] Ghosh S, Kumar S, Chaudhary R, Guha P (2023). High-risk human papillomavirus infection in squamous cell carcinoma of the larynx: a study from a tertiary care center in North India. Cureus.

[ref-11] Gluvajic D, Hosnjak L, Stegel V, Novakovic S, Gale N, Poljak M, Boltezar IH (2020). Risk factors for the development of high-grade dysplasia and carcinoma in patients with laryngeal squamous cell papillomas: large retrospective cohort study. Head & Neck.

[ref-12] Han Y, Wang X, Xia K, Su T (2021). A novel defined hypoxia-related gene signature to predict the prognosis of oral squamous cell carcinoma. Annals of Translational Medicine.

[ref-13] Hefni E, Menon D, Ma T, Asiedu EB, Sultan A, Meiller T, Schneider A, Sodhi A, Montaner S (2023). Angiopoietin-like 4 induces head and neck squamous cell carcinoma cell migration through the NRP1/ABL1/PXN pathway. Celllular Signalling.

[ref-14] Hsieh CY, Lin CC, Huang YW, Chen JH, Tsou YA, Chang LC, Fan CC, Lin CY, Chang WC (2022). Macrophage secretory IL-1beta promotes docetaxel resistance in head and neck squamous carcinoma via SOD2/CAT-ICAM1 signaling. JCI Insight.

[ref-15] Jemal A, Siegel R, Ward E, Murray T, Xu J, Thun MJ (2007). Cancer statistics, 2007. CA: A Cancer Journal for Clinicians.

[ref-16] Johnson DE, Burtness B, Leemans CR, Lui VWY, Bauman JE, Grandis JR (2020). Head and neck squamous cell carcinoma. Nature Reviews Disease Primers.

[ref-17] Kunkel M, Reichert TE, Benz P, Lehr HA, Jeong JH, Wieand S, Bartenstein P, Wagner W, Whiteside TL (2003). Overexpression of Glut-1 and increased glucose metabolism in tumors are associated with a poor prognosis in patients with oral squamous cell carcinoma. Cancer.

[ref-18] Kwon EJ, Lee HR, Lee JH, Seo C, Ha M, Roh J, Kim YH, Jang JY (2022). Identification of differentially expressed genes and pathways for risk stratification in HPV-associated cancers governing different anatomical sites. Frontiers in Bioscience (Landmark Edition).

[ref-19] Li B, Hao Y, He H, Fan Y, Ren B, Peng X, Zhou X, Chen L (2024a). CD47-SIRPα blockade sensitizes head and neck squamous cell carcinoma to cetuximab by enhancing macrophage adhesion to cancer cells. Cancer Research.

[ref-20] Li J, Tan J, Wang T, Yu S, Guo G, Li K, Yang L, Zeng B, Mei X, Gao S, Lao X, Zhang S, Liao G, Liang Y (2024b). cGAS-ISG15-RAGE axis reprogram necroptotic microenvironment and promote lymphatic metastasis in head and neck cancer. Experimental Hematology & Oncology.

[ref-21] Liberale C, Soloperto D, Marchioni A, Monzani D, Sacchetto L (2023). Updates on larynx cancer: risk factors and oncogenesis. International Journal of Molecular Sciences.

[ref-22] Liu M, Zhang Y, Yang J, Cui X, Zhou Z, Zhan H, Ding K, Tian X, Yang Z, Fung KA, Edil BH, Postier RG, Bronze MS, Fernandez-Zapico ME, Stemmler MP, Brabletz T, Li YP, Houchen CW, Li M (2020). ZIP4 increases expression of transcription factor ZEB1 to promote integrin alpha3beta1 signaling and inhibit expression of the gemcitabine transporter ENT1 in pancreatic cancer cells. Gastroenterology.

[ref-23] Love MI, Huber W, Anders S (2014). Moderated estimation of fold change and dispersion for RNA-seq data with DESeq2. Genome Biology.

[ref-24] Lv T, Liu H, Mao L, Song Y, Liao L, Zhong K, Shuai B, Luo Y, Guo T, Huang W, Zhang S (2024). Cancer-associated fibroblast-derived extracellular vesicles promote lymph node metastases in oral cavity squamous cell carcinoma by encapsulating ITGB1 and BMI1. BMC Cancer.

[ref-25] Mathew M, Nguyen NT, Bhutia YD, Sivaprakasam S, Ganapathy V (2024). Metabolic signature of warburg effect in cancer: an effective and obligatory interplay between nutrient transporters and catabolic/anabolic pathways to promote tumor growth. Cancers.

[ref-26] Miller ZA, Muthuswami S, Mueller A, Ma RZ, Sywanycz SM, Naik A, Huang L, Brody RM, Diab A, Carey RM, Lee RJ (2024). GLUT1 inhibitor BAY-876 induces apoptosis and enhances anti-cancer effects of bitter receptor agonists in head and neck squamous carcinoma cells. Cell Death Discovery.

[ref-27] Okada R, Goto Y, Yamada Y, Kato M, Asai S, Moriya S, Ichikawa T, Seki N (2020). Regulation of oncogenic targets by the tumor-suppressive miR-139 duplex (miR-139-5p and miR-139-3p) in renal cell carcinoma. Biomedicines.

[ref-28] Palollathil A, Babu S, Abhinand CS, Mathew RT, Vijayakumar M, Prasad TSK (2024). Proteomic profiling of oral squamous cell carcinoma tissues reveals altered immune-related proteins: implications for personalized therapy. Expert Review of Proteomics.

[ref-29] Panuganti BA, Finegersh A, Flagg M, Tu X, Orosco R, Weissbrod PA, Califano J (2021). Prognostic significance of HPV status in laryngeal squamous cell carcinoma: a large-population database study. Otolaryngology–Head and Neck Surgery.

[ref-30] Park SJ, Min HJ, Yoon C, Kim SH, Kim JH, Lee SY (2023). Integrin beta1 regulates the perineural invasion and radioresistance of oral squamous carcinoma cells by modulating cancer cell stemness. Cellular Signalling.

[ref-31] Pinkiewicz M, Dorobisz K, Zatonski T (2022). Human papillomavirus-associated head and neck cancers. Cancer Management and Research.

[ref-32] Qin Y, Li Z, Liu T, Ma J, Liu H, Zhou Y, Wang S, Zhang L, Peng Q, Ye P, Duan N, Wang W, Wang X (2024). Prevotella intermedia boosts OSCC progression through ISG15 upregulation: a new target for intervention. Journal of Cancer Research and Clinical Oncology.

[ref-33] Sherman BT, Hao M, Qiu J, Jiao X, Baseler MW, Lane HC, Imamichi T, Chang W (2022). DAVID: a web server for functional enrichment analysis and functional annotation of gene lists (2021 update). Nucleic Acids Research.

[ref-34] Stumbryte-Kaminskiene A, Gudleviciene Z, Dabkeviciene D, Mackeviciene I (2020). Combined effect of HPV and several gene SNPs in laryngeal cancer. Medicina.

[ref-35] Swartz JE, Pothen AJ, van Kempen PM, Stegeman I, Formsma FK, Cann EM, Willems SM, Grolman W (2016). Poor prognosis in human papillomavirus-positive oropharyngeal squamous cell carcinomas that overexpress hypoxia inducible factor-1alpha. Head & Neck.

[ref-36] Thakore VP, Patel KD, Vora HH, Patel PS, Jain NK (2024). Up-regulation of extracellular-matrix and inflammation related genes in oral squamous cell carcinoma. Archives of Oral Biology.

[ref-37] Wang Y, Ding X, Liu B, Li M, Chang Y, Shen H, Xie SM, Xing L, Li Y (2020). ETV4 overexpression promotes progression of non-small cell lung cancer by upregulating PXN and MMP1 transcriptionally. Molecular Carcinogenesis.

[ref-38] Wang P, Wang YY, Xu YL, Zhang CY, Wang K, Wang Q (2023). Phosphoglycerate-kinase-1 is a potential prognostic biomarker in HNSCC and correlates with immune cell infiltration. Cancer Genomics & Proteomics.

[ref-39] Wu DW, Chuang CY, Lin WL, Sung WW, Cheng YW, Lee H (2014). Paxillin promotes tumor progression and predicts survival and relapse in oral cavity squamous cell carcinoma by microRNA-218 targeting. Carcinogenesis.

[ref-40] Wu P, Hou X, Peng M, Deng X, Yan Q, Fan C, Mo Y, Wang Y, Li Z, Wang F, Guo C, Zhou M, Liao Q, Wang H, Zeng Z, Jiang W, Li G, Xiong W, Xiang B (2023). Circular RNA circRILPL1 promotes nasopharyngeal carcinoma malignant progression by activating the Hippo-YAP signaling pathway. Cell Death & Differentiation.

[ref-41] Xie J, Guo T, Zhong Z, Wang N, Liang Y, Zeng W, Liu S, Chen Q, Tang X, Wu H, Zhang S, Ma K, Wang B, Ou Y, Gu W, Chen H, Qiu Y, Duan Y (2021). ITGB1 drives hepatocellular carcinoma progression by modulating cell cycle process through PXN/YWHAZ/AKT pathways. Frontiers in Cell and Developmental Biology.

[ref-42] Zhang C, Deng Z, Chen Y, Suzuki M, Xie M (2016). Is there a higher prevalence of human papillomavirus infection in Chinese laryngeal cancer patients? a systematic review and meta-analysis. European Archives of Oto-Rhino-Laryngology.

[ref-43] Zhang Y, Li J, Lai XN, Jiao XQ, Xiong JP, Xiong LX (2019). Focus on Cdc42 in breast cancer: new insights, target therapy development and non-coding RNAs. Cells.

[ref-44] Zhao Q, Zhang Y, Zhang X, Sun Y, Lin Z (2020). Mining of gene modules and identification of key genes in head and neck squamous cell carcinoma based on gene co-expression network analysis. Medicine.

[ref-45] Zolkind P, Przybylski D, Marjanovic N, Nguyen L, Lin T, Johanns T, Alexandrov A, Zhou L, Allen CT, Miceli AP, Schreiber RD, Artyomov M, Dunn GP, Uppaluri R (2017). Cancer immunogenomic approach to neoantigen discovery in a checkpoint blockade responsive murine model of oral cavity squamous cell carcinoma. Oncotarget.

