# Supplementary material for: Bioinformatics analysis of laryngeal squamous cell carcinoma based on the high infection rate of HPV in Northwest China
Source: PeerJ. 2025 Aug 11;13:e19851. doi: 10.7717/peerj.19851 (PMC12352419; doi:10.7717/peerj.19851)
Supplement: Supplemental Information 5 [file peerj-13-19851-s005.docx]

| **KEGG ID** | **Term** | **Count** | **Enriched proteins** | **%** | **P-Value** |
| --- | --- | --- | --- | --- | --- |
| hsa05205 | Proteoglycans in cancer | 5 | CDC42, ITGB1, PXN, FGF2, EGFR | 45.5 | 0.000052 |
| hsa04810 | Regulation of actin cytoskeleton | 5 | CDC42, ITGB1, PXN, FGF2, EGFR | 45.5 | 0.000083 |
| hsa04066 | HIF-1 signaling pathway | 4 | SLC2A1, PGK1, GAPDH, EGFR | 36.4 | 0.00021 |
| hsa04670 | Leukocyte transendothelial migration | 4 | CDC42, ITGB1, PXN, ICAM1 | 36.4 | 0.00025 |
| hsa05165 | Human papillomavirus infection | 5 | CDC42, ITGB1, PXN, ISG15, EGFR | 45.5 | 0.00035 |
| hsa04510 | Focal adhesion | 4 | CDC42, ITGB1, PXN, EGFR | 36.4 | 0.0013 |
| hsa04015 | Rap1 signaling pathway | 4 | CDC42, ITGB1, FGF2, EGFR | 36.4 | 0.0014 |
| hsa05200 | Pathways in cancer | 5 | CDC42, ITGB1, SLC2A1, FGF2, EGFR | 45.5 | 0.002 |
| hsa05131 | Shigellosis | 4 | CDC42, ITGB1, PXN, EGFR | 36.4 | 0.0023 |
| hsa05100 | Bacterial invasion of epithelial cells | 3 | CDC42, ITGB1, PXN | 27.3 | 0.0033 |
| hsa05135 | Yersinia infection | 3 | CDC42, ITGB1, PXN | 27.3 | 0.01 |
| hsa05130 | Pathogenic Escherichia coli infection | 3 | CDC42, ITGB1, GAPDH | 27.3 | 0.021 |
| hsa04014 | Ras signaling pathway | 3 | CDC42, FGF2, EGFR | 27.3 | 0.028 |
| hsa04010 | MAPK signaling pathway | 3 | CDC42, FGF2, EGFR | 27.3 | 0.043 |
