# Supplementary material for: Bioinformatics analysis of laryngeal squamous cell carcinoma based on the high infection rate of HPV in Northwest China
Source: PeerJ. 2025 Aug 11;13:e19851. doi: 10.7717/peerj.19851 (PMC12352419; doi:10.7717/peerj.19851)
Supplement: Supplemental Information 7 [file peerj-13-19851-s007.docx]

**Sample Name**: TU212
**Detection Method**: DNA was extracted using Axygen's genomic extraction kit. A 20-STR amplification protocol was employed for amplification. The STR loci and the sex gene Amelogenin were detected on an ABI 3730XL genetic analyzer.

**Detection Results**: The DNA typing of this cell line was found to have a perfect match in the cell line search. The EXPASY database indicates that the cell name is Tu 212, and the cell number corresponds to CVCL_4915. No multi-alleles were detected in this cell line during this test. Cell match value: 1.0. Problematic cell line: Contaminated. T404, T406, Tu 138, Tu 158LN, Tu 159, Tu 182, Tu 212, and Tu 212LN have been shown to be identical (PubMed=21868764).

**STR Database Comparison Analysis**: The genotyping results of the STR loci and the Amelogenin locus of the tested cells were compared with the STR data of 2,455 cell lines 收录于 ExPASY, ATCC, DSMZ, JCRB, and RIKEN databases. If the tested cells are not 收录于 the above cell banks or are newly established cell lines, no comparison can be made. Users need to compare the cell typing results with other databases by themselves.

**Typing Results**:

| Loci | STR Information of Submitted Cells | | | STR Information of Cells in the Database | | |
| --- | --- | --- | --- | --- | --- | --- |
|  | Submitted Cell Name: TU212 | | | Cell Name in the Database: TU212 | | |
|  | Allele1 | Allele2 | Allele3 | Allele1 | Allele2 | Allele3 |
| D5S818 | 12 | 13 |  | 12 | 13 |  |
| D13S317 | 12 | 12 |  | 12 | 12 |  |
| D7S820 | 10 | 10 |  | 10 | 10 |  |
| D16S539 | 8 | 9 |  | 8 | 9 |  |
| VWA | 15 | 16 |  | 15 | 16 |  |
| TH01 | 8 | 8 |  | 8 | 8 |  |
| AMEL | X | X |  | X | X |  |
| TPOX | 10 | 11 |  | 10 | 11 |  |
| CSF1PO | 12 | 13 |  | 12 | 13 |  |
| D12S391 | 17 | 19 |  |  |  |  |
| FGA | 18.2 | 18.2 |  |  |  |  |
| D2S1338 | 21 | 21 |  |  |  |  |
| D21S11 | 28 | 29 |  |  |  |  |
| D18S51 | 14 | 14 |  |  |  |  |
| D8S1179 | 13 | 13 |  |  |  |  |
| D3S1358 | 14 | 14 |  |  |  |  |
| D6S1043 | 15 | 17 |  |  |  |  |
| PENTAE | 12 | 12 |  |  |  |  |
| D19S433 | 13 | 13 |  |  |  |  |
| PENTAD | 14 | 14 |  |  |  |  |
| D1S1656 | 13 | 16.3 |  |  |  |  |


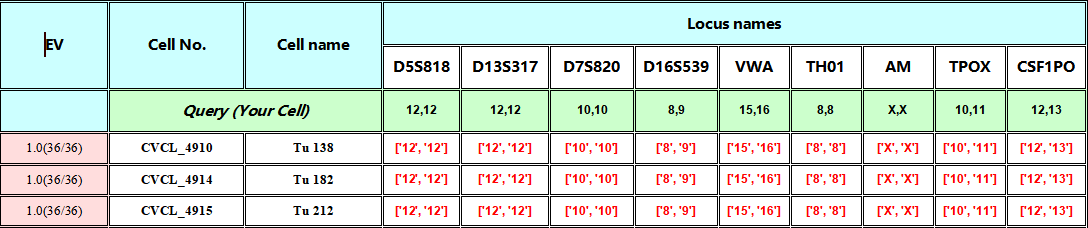


**Typing Map**:


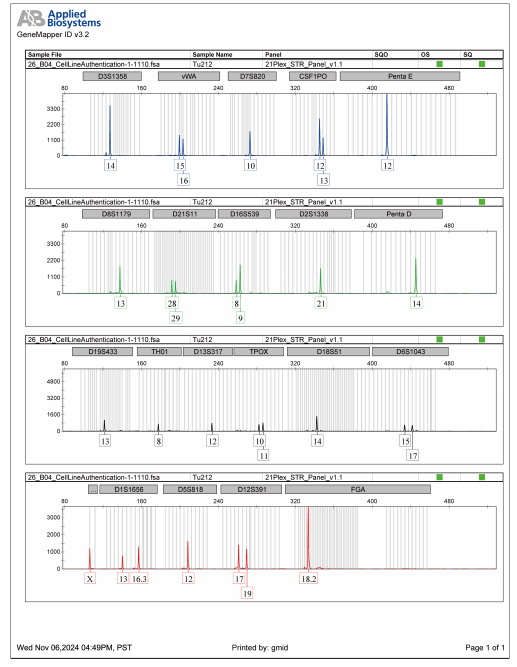


**Remarks**:

1. According to the cell STR identification standard formulated by the International Cell Line Authentication Committee (ICLAC), if the match degree is ≥ 80%, the cell line can be considered correct. If the match degree is < 80%, the origin of the cell line needs to be suspected.
2. The effective peak positions in the map are real PCR bands. Small peaks and non-specific bands are ignored in the calculation.
3. National Experimental Cell Resource Sharing Platform, database entry: <http://www.cellresource.cn/>.
