# Supplementary figures and images for: Bioinformatics analysis of laryngeal squamous cell carcinoma based on the high infection rate of HPV in Northwest China
Source: PeerJ. 2025 Aug 11;13:e19851. doi: 10.7717/peerj.19851 (PMC12352419; doi:10.7717/peerj.19851)

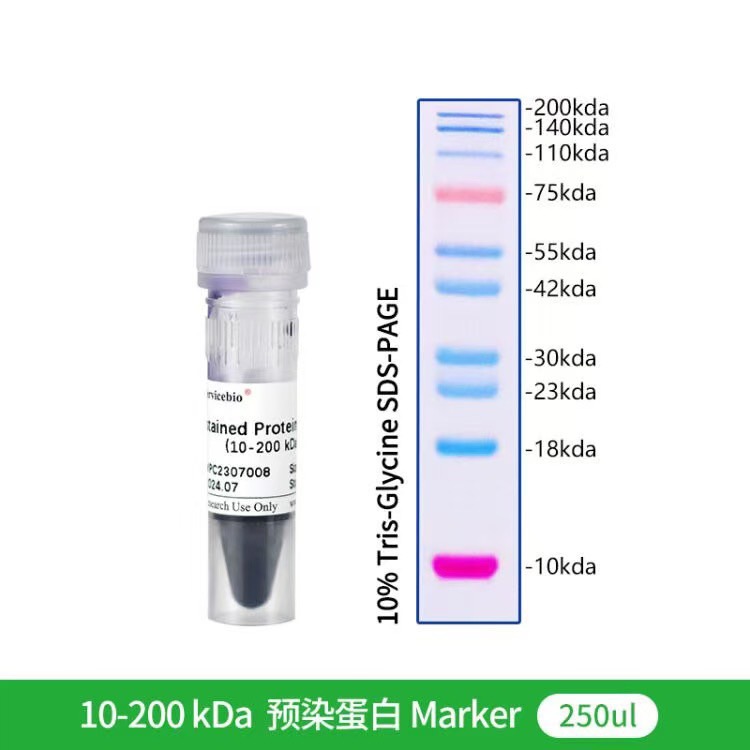

Supplement: Supplemental Information 9 — Full-length uncropped gels/blots. A photograph of the reassembled pieces to confirm that they come from the same original full-length blot. Molecular weight ladders, controls and reference samples must be visible. [file peerj-13-19851-s009.zip › HPV16E7 WB original data/maker.JPG]

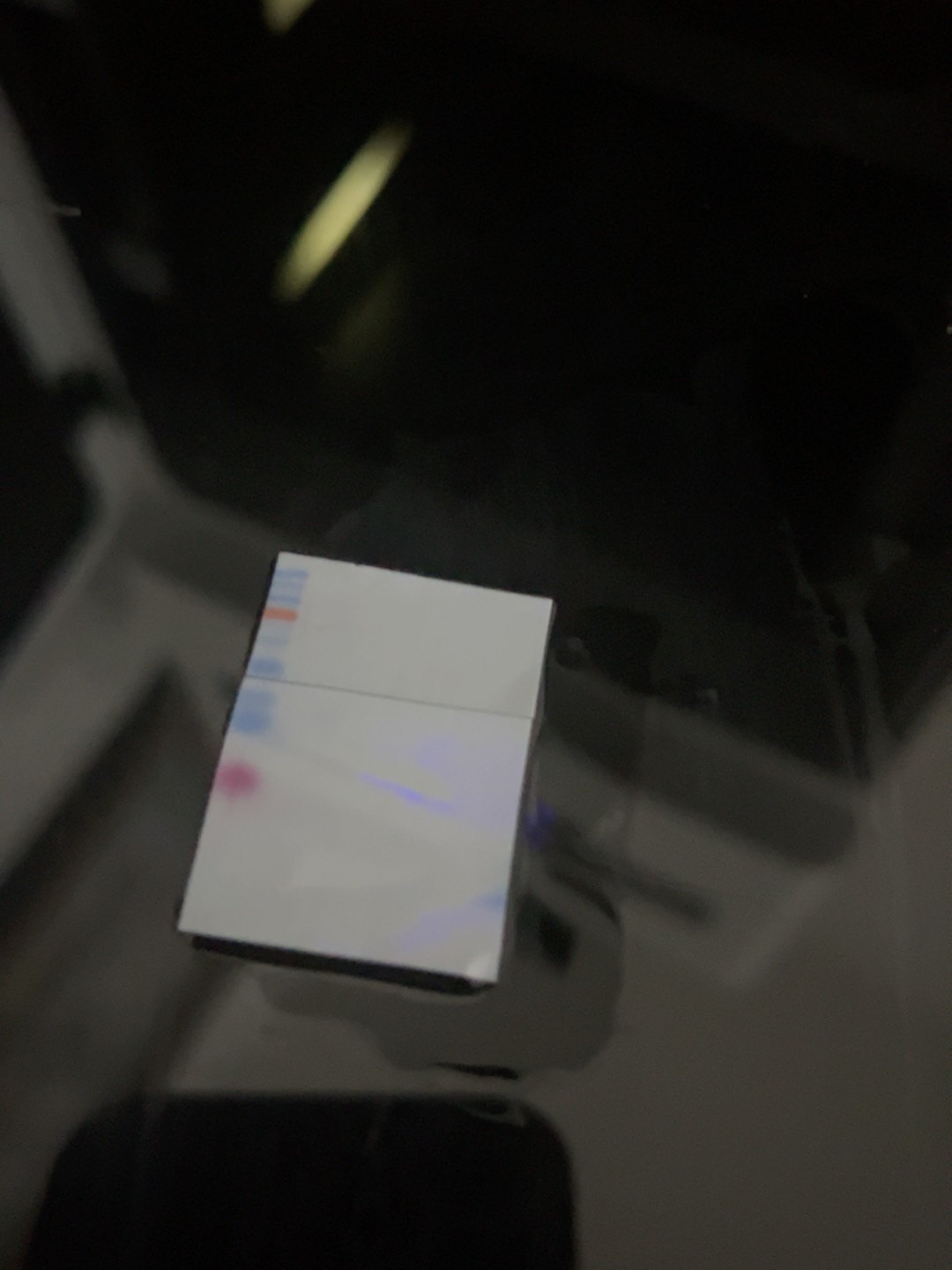

Supplement: Supplemental Information 9 — Full-length uncropped gels/blots. A photograph of the reassembled pieces to confirm that they come from the same original full-length blot. Molecular weight ladders, controls and reference samples must be visible. [file peerj-13-19851-s009.zip › HPV16E7 WB original data/clipped graph.JPG]

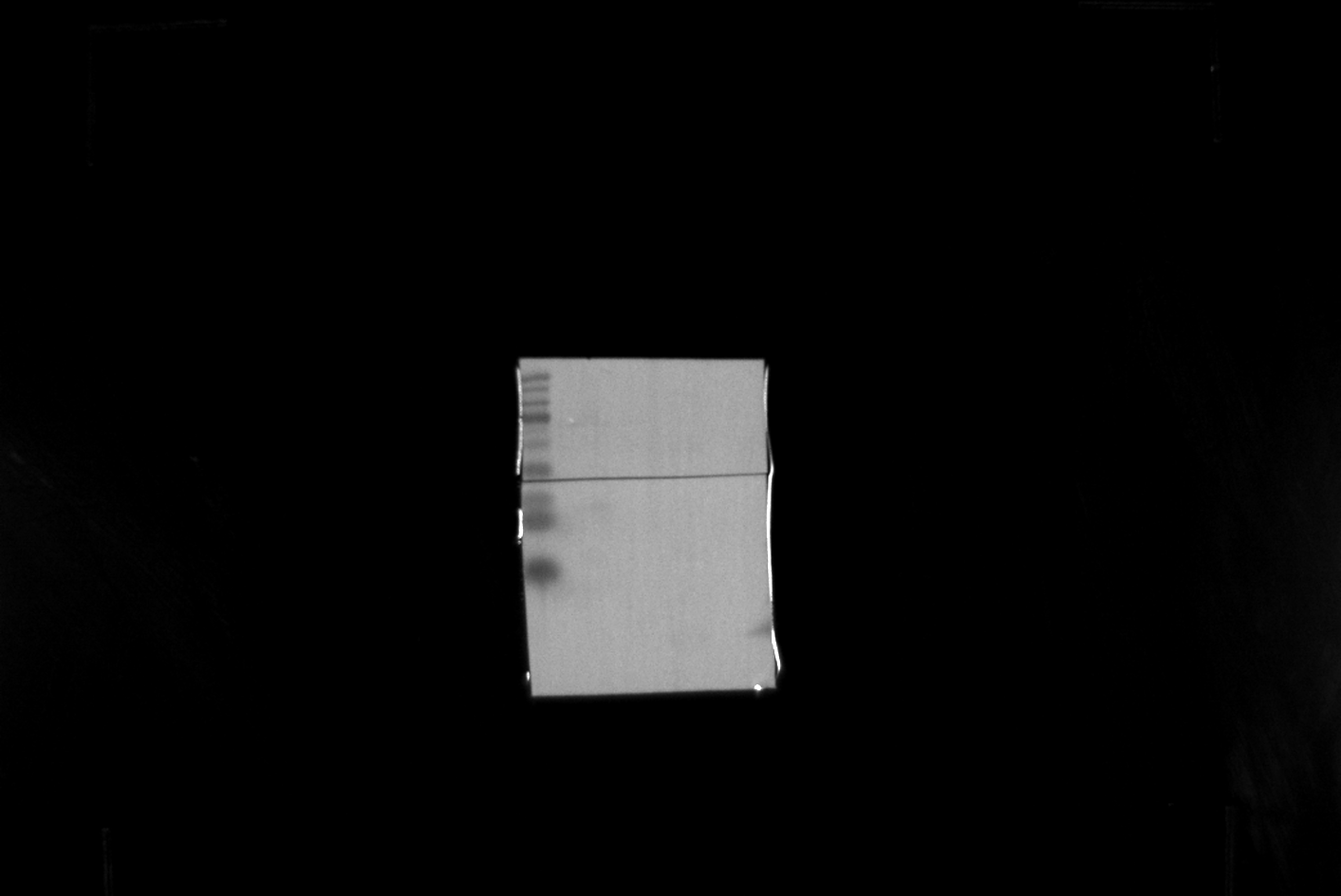

Supplement: Supplemental Information 9 — Full-length uncropped gels/blots. A photograph of the reassembled pieces to confirm that they come from the same original full-length blot. Molecular weight ladders, controls and reference samples must be visible. [file peerj-13-19851-s009.zip › HPV16E7 WB original data/HPV16E7 WB white - field image.tif]

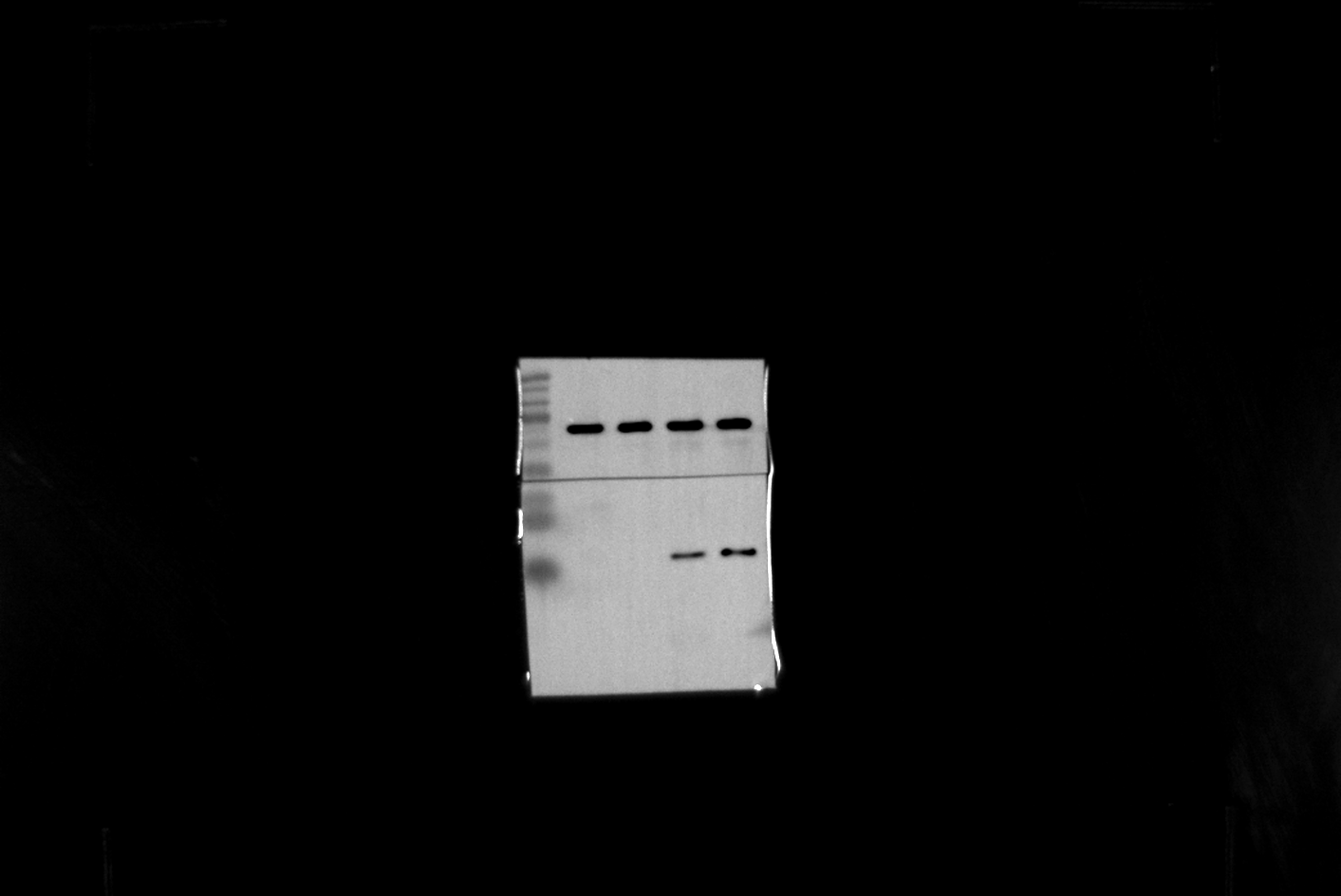

Supplement: Supplemental Information 9 — Full-length uncropped gels/blots. A photograph of the reassembled pieces to confirm that they come from the same original full-length blot. Molecular weight ladders, controls and reference samples must be visible. [file peerj-13-19851-s009.zip › HPV16E7 WB original data/HPV16E7 merge.tif]

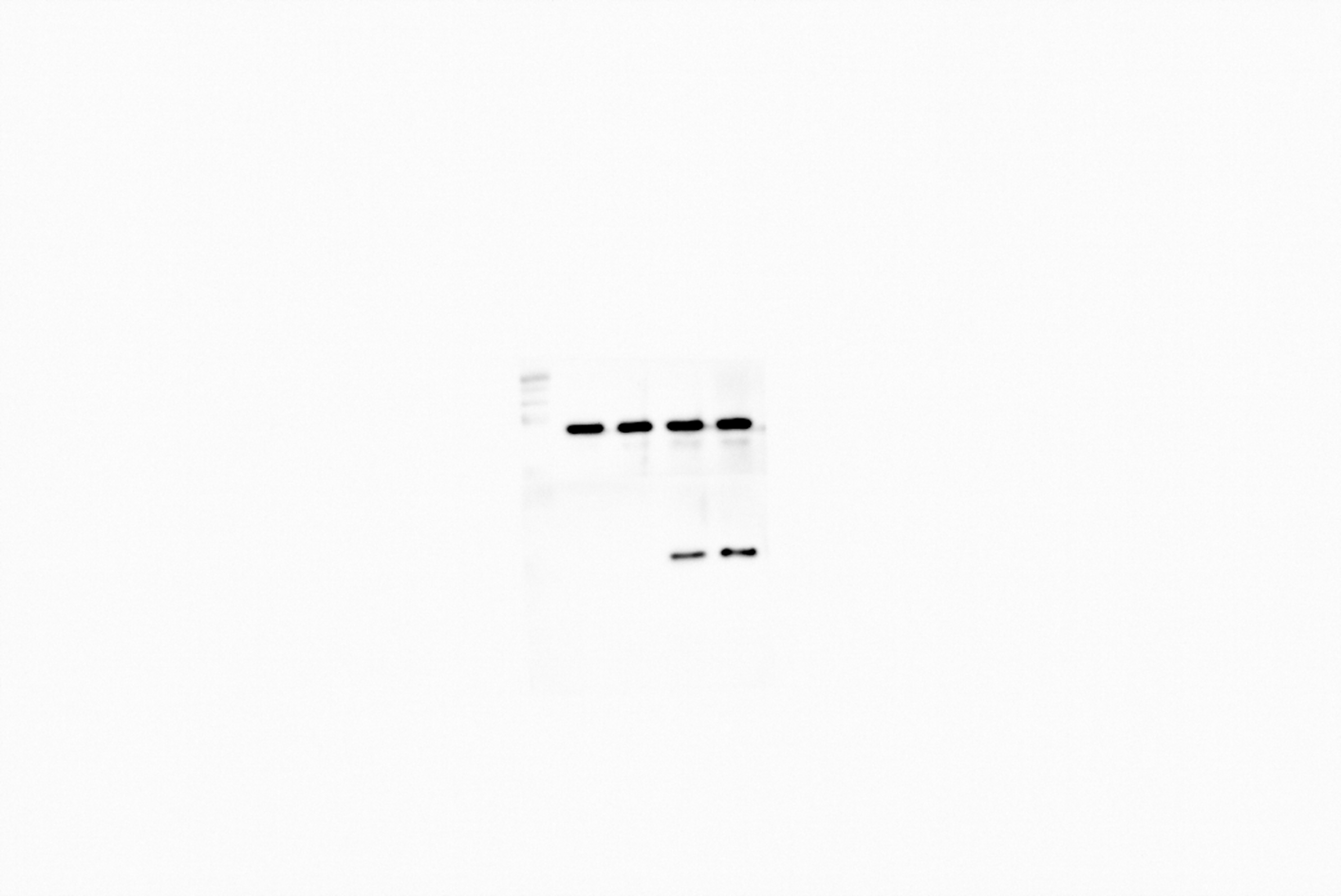

Supplement: Supplemental Information 9 — Full-length uncropped gels/blots. A photograph of the reassembled pieces to confirm that they come from the same original full-length blot. Molecular weight ladders, controls and reference samples must be visible. [file peerj-13-19851-s009.zip › HPV16E7 WB original data/HPV16E7 WB Band graph.tif]

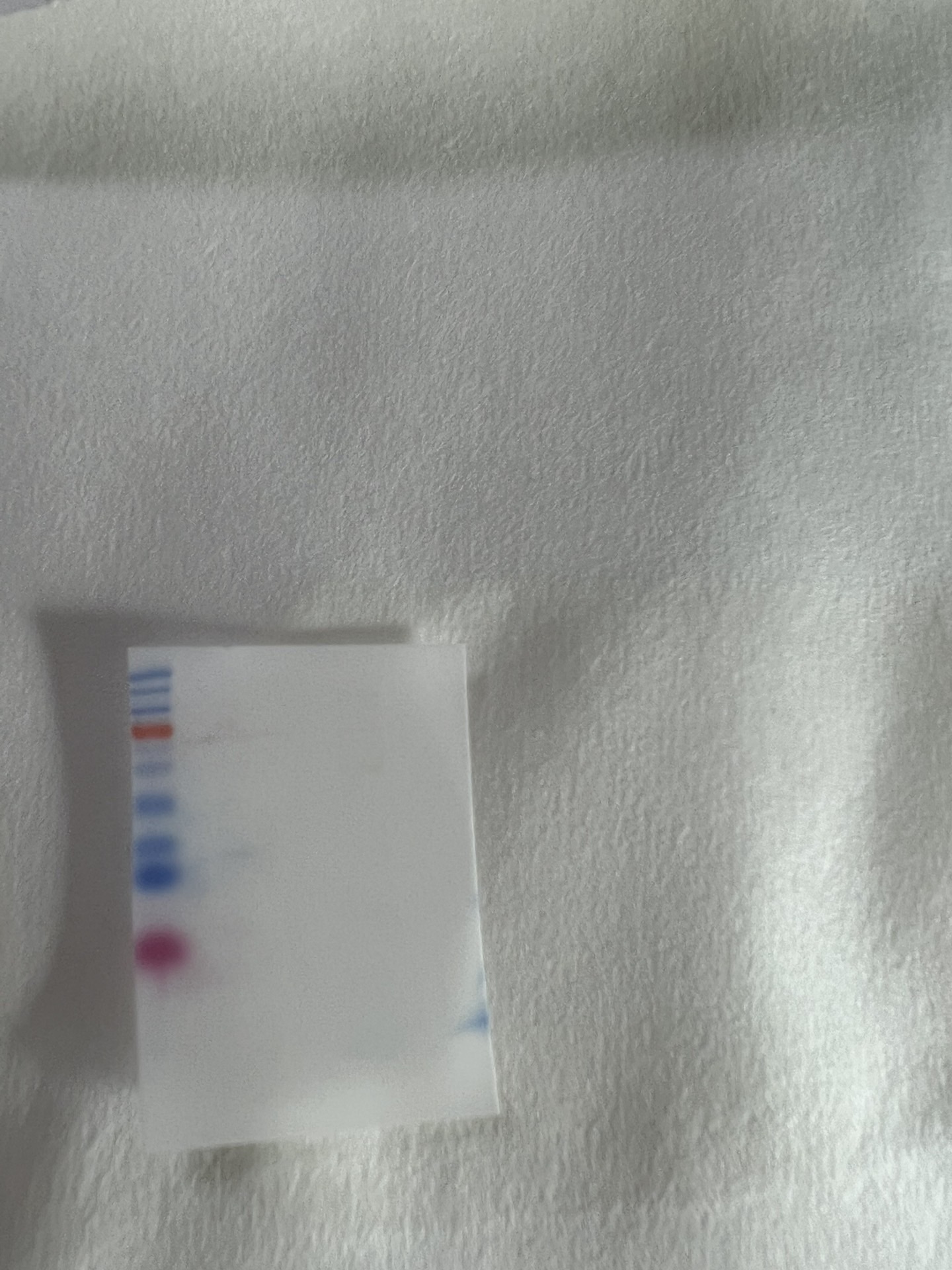

Supplement: Supplemental Information 9 — Full-length uncropped gels/blots. A photograph of the reassembled pieces to confirm that they come from the same original full-length blot. Molecular weight ladders, controls and reference samples must be visible. [file peerj-13-19851-s009.zip › HPV16E7 WB original data/Unclipped graph.JPG]
